# Newborn Hearing Screenings in Human Immunodeficiency Virus-Exposed Uninfected Infants

**Published:** 2016-09-05

**Authors:** P Torre, B Zeldow, TJ Yao, HJ Hoffman, GK Siberry, MU Purswani, T Frederick, SA Spector, PL Williams

**Affiliations:** 1 School of Speech, Language, and Hearing Sciences, San Diego State University, San Diego, CA, USA; 2 Center for Biostatistics in AIDS Research, Harvard T. H. Chan School of Public Health, Boston, MA; 3 Epidemiology and Statistics Program, National Institute on Deafness and Other Communication Disorders, National Institutes of Health, Bethesda, MD, USA; 4 Eunice Kennedy Shriver National Institute of Child Health and Human Development, National Institutes of Health, Bethesda, MD, USA; 5 Albert Einstein College of Medicine, Department of Pediatric Infectious Disease, Bronx-Lebanon Hospital Center, Bronx, NY, USA; 6 Department of Maternal, Child & Adolescent Center for Infectious Diseases and Virology, University of Southern California, Alhambra, CA, USA; 7 Department of Pediatrics, University of California San Diego, La Jolla, CA and Rady Children's Hospital San Diego, CA, USA

**Keywords:** Newborn, Hearing, Human Immunodeficiency Virus, Cytomegalovirus, In Utero Antiretroviral Exposure

## Abstract

Perinatal HIV infection and congenital cytomegalovirus (CMV) infection may increase the risk for hearing loss. We examined 1,435 infants enrolled in the Surveillance Monitoring of ART Toxicities (SMARTT) study of the Pediatric HIV/AIDS Cohort Study (PHACS) network, a prospective study of the safety of *in utero* antiretroviral (ARV) exposures. We determined the proportion of perinatally HIV-exposed uninfected (HEU) newborns who were referred for additional hearing testing, and evaluated the association between *in utero* ARV exposures and newborn hearing screening results. Using a nested case-control design, we also examined congenital CMV infection in infants with and without screening referral. Congenital CMV infection was determined based on CMV DNA detection using a nested PCR assay in peripheral blood mononuclear cells obtained within 14 days of birth. Among the 1,435 infants (70% black, 31% Hispanic, 51% male), 45 (3.1%) did not pass the hearing screen and were referred for further hearing testing. Based on exact logistic regression models controlling for maternal use of tobacco and ototoxic medications, first trimester exposure to Tenofovir was associated with lower odds of a newborn hearing screening referral [adjusted odds ratio (aOR) = 0.41, 95% confidence interval (CI): 0.14-1.00]. Exposure to Atazanavir was linked to higher odds of newborn screening referral, although not attaining significance [aOR = 1.84, 95% CI: 0.92-3.56]. Maternal ARV use may have varying effects on newborn hearing screenings. These results highlight the importance for audiologists to be knowledgeable of *in utero* ARV exposures in HEU children because of the possibility of higher referrals in these children.

## Introduction

Universal newborn hearing screening in the United States was implemented following the 1993 NIH Consensus Development Conference that endorsed the screening for hearing loss of all newborns before leaving the hospital [1]. This landmark move led to an unprecedented state-by-state effort to promote mandatory newborn screening, which was bolstered by Congressional passage of the Newborn and Infant Hearing Screening and Intervention Act of 1999 that provided funding for statewide programs [2,3]. Late identification of permanent hearing loss can impair long-term speech and language development, and, subsequently, educational achievement [4,5]. The overall rate of permanent hearing loss has been reported as approximately 2 per 1000 live births [6,7]. Human immunodeficiency virus (HIV) is a risk factor for hearing loss in children and adolescents [8], but the potential link between hearing loss and *in utero* exposure to maternal HIV infection and HIV medications has not been well studied.

Studies of hearing screening results obtained from babies born to mothers infected with HIV are limited. Two studies have observed about two-fold higher risk of hearing loss for HIV-exposed uninfected (HEU) infants as compared to HIV-unexposed and uninfected (HUU) infants, although neither finding attained statistical significance due to the low prevalence of hearing loss [9,10]. In addition, neither accounted for congenital cytomegalovirus (CMV), which is well-known to be linked to sensorineural hearing loss [11-14] and has a higher prevalence in neonates born to mothers with HIV [15,16]. While the prevalence of congenital CMV in HEU infants has decreased with the advent of highly active antiretroviral therapy (HAART), it remains higher than in the general population [17].

Early identification of newborn hearing loss has important implications for the child's speech, language and educational development. Since congenital CMV infection has been identified as a risk factor for permanent sensorineural hearing loss [13], screening for CMV co-infection in this group of infants is important in understanding hearing loss in HEU. The objectives of this project were to: 1) determine the proportion of HEU children referred for additional testing following a newborn hearing screening in the Surveillance Monitoring of ART Toxicities (SMARTT) cohort study (a protocol within the Pediatric HIV/AIDS Cohort Study [PHACS]); 2) evaluate the association of *in utero* antiretroviral (ARV) exposures with newborn hearing screening results; 3) evaluate the association between newborn hearing screening results and other risk factors and 4) examine congenital CMV infection in a subset of infants with and without further hearing referrals as a result of the newborn screening.

## Materials and Methods

The study population was the Dynamic Cohort of the SMARTT study which prospectively enrolls pregnant women and their newborns from week 22 of gestation through 72 hours after birth, and follows both the mothers and their children annually. The SMARTT study is a US based, multisite, prospective cohort study designed to evaluate the effects of *in utero* antiretroviral (ARV) exposure on outcomes across several domains among HEU infants (for additional details of the SMARTT study design, see Williams et al. [18]. Newborn hearing screening and follow-up screening results were collected from the medical record and then reported to the SMARTT study on study-specific data collection forms. Hearing screenings included otoacoustic emission (OAE) measures, automated auditory brainstem response (AABR) measures, or both depending on the hospital protocol. The primary outcome for this analysis was the hearing screening result (pass or refer) using OAE or AABR.

Maternal ARV data were retrospectively collected for the entire pregnancy through chart review or from prior studies; information collected included start and stop dates of each individual ARV drug so that both duration and timing of exposure (by trimester) could be characterized. The primary exposures of interest were *in utero* ARV exposure overall during pregnancy and within individual trimesters. HAART was considered as any regimen containing at least three drugs from at least two different drug classes. Regimens that included three nucleoside reverse transcriptase inhibitors (NRTIs) were also evaluated, as were individual drug classes (NRTI, non-NRTI, and protease inhibitors) and individual ARV agents. Demographic characteristics and maternal and birth characteristics were collected by questionnaire and medical record review and treated as possible risk factors. Substance use during pregnancy was self-reported by mothers as reported previously [19].

A nested sub-study within SMARTT was conducted to address the issue of congenital CMV, since the SMARTT study itself did not determine CMV infection status on all newborns. Congenital CMV infection was determined in a subset of participants based on CMV DNA detection in peripheral blood mononuclear cells (PBMCs) drawn at the entry study visit (usually at birth) and stored in the PHACS repository using a sensitive nested PCR assay [20]. Samples were chosen in a case-control design with four samples (subject to sample availability) from infants who passed the newborn hearing screen for every one infant who was referred for further testing.

Demographic differences between infants who passed and those who were referred for further hearing testing were examined using chi-square or Wilcoxon tests, as appropriate. The prevalence of hearing screening referral and its exact 95% confidence interval (CI) were calculated under a binomial distribution. Due to the relatively rare outcome of not passing a newborn hearing screening, exact logistic regression models were used to evaluate the association of maternal ARV exposures with newborn hearing outcomes. Adjusted models included a priori identified confounders with a *p-value* < 0.15 in unadjusted analyses (Supplemental Table 1).

## Results

As of January 1, 2013, of 1,435 SMARTT infants were enrolled and had newborn hearing screening data. The infants were primarily black (70%), with 31% Hispanic and 51% male. Among these 1,435 infants, 45 (3.1%, exact 95% CI: 2.3% - 4.2%) did not pass the newborn hearing screening and were referred for further testing. Of the 1,435 infants, 1,406 (98%) had detailed information on child demographics and maternal characteristics (Table 1) and subsequent analyses were restricted to these 1406 infants. Almost all infants (1,382 or 98%) were exposed to either HAART or a triple NRTI regimen, with 707 (50%) exposed in the first trimester. The most common ARV exposures (Supplemental Table 2) were lamivudine (67%) and zidovudine (66%); 43% were exposed to Tenofovir, 24% to Atazanavir, and 5% to efavirenz. Only 17 infants (1%) had no recorded ARV exposure.

**Figure 1:**
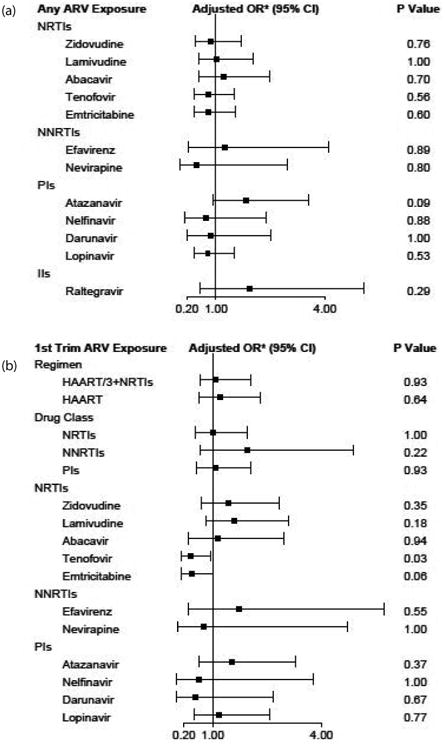
Estimated associations between maternal ARV exposures and newborn hearing screening referral (or failed screening) using exact adjusted logistic regression, separated by any exposure (1a) 1^st^ trimester exposure (1b) Associations were adjusted for maternal use of ototoxic medications and in utero tobacco exposure.

Based on unadjusted exact logistic regression models, first trimester exposure to Tenofovir was associated with lower odds of a newborn hearing screening referral [odds ratio (OR) = 0.41, 95% CI 0.14-1.00, *p = 0.049*], and first trimester exposure to emtricitabine was also associated with lower odds of a referral (OR = 0.45, 95% CI 0.15-1.07, *p = 0.08*), although not attaining statistical significance (Figure 1). After controlling for maternal use of tobacco and ototoxic medications, the association between Tenofovir and screening referral remained significant [adjusted OR (aOR) = 0.39, 95% CI 0.39-0.94, *p* = *0.03*] and the association between emtricitabine and screening referral remained similar (Figure 1). Exposure to Atazanavir during pregnancy was associated with higher odds of newborn screening referral (aOR = 1.84, 95% CI 0.92-3.56, *p = 0.09*); in addition, a similar magnitude of association was observed for infants with third trimester exposure to Atazanavir (aOR = 1.92, 95% CI 0.94-3.76, *p = 0.07*; data not shown). There were no significant associations of newborn screening results with either demographic characteristics (including sex, ethnicity, and race) or maternal and birth characteristics (including mother's age, alcohol, tobacco, or ototoxic medication use during pregnancy, birth weight, or gestation age).

Among the 45 infants referred after the initial newborn hearing screening, 22 had acceptable PBMC samples for CMV testing and one (4.5%) was CMV-positive. Of the 1,390 infants who passed the hearing screening, 92 were tested and 5 (5.4%) were CMV-positive. Follow-up hearing testing was not available for the six CMV positive infants.

Follow-up information on subsequent hearing tests for the 45 infants referred for further testing was evaluated through January 1, 2013. Median age at follow-up was three years with the oldest child under six years. Twelve of the 45 (27%) infants did not receive any follow-up hearing testing. Twenty-four (73%) of the remaining 33 infants with subsequent follow-up passed their hearing screenings. Another nine infants (27%) continued to have some degree of hearing problems on additional screenings or testing, including one (3%) with confirmed sensorineural hearing loss after an extensive hearing examination.

## Discussion

Exposure to Atazanavir overall and specifically in the third trimester was linked with higher odds of a referral, but this association did not reach statistical significance. A recent report from within the same cohort found a significant association between exposure to Atazanavir and delayed language developmental [21]. In that analysis, the significantly higher odds increased further if the exposure to Atazanavir occurred after the first trimester. This potential relationship between perinatal exposure to Atazanavir and language delay and possibly hearing problems requires further study.

Conversely, exposure to Tenofovir in the first trimester was associated with significantly lower odds of a referral for further hearing testing. Further, first trimester exposure to emtricitabine, typically given in tablets co-formulated with Tenofovir was also associated with lower odds of a referral, but this did not attain statistical significance.

Alternative approaches for evaluating associations between exposures and binary outcomes in the setting of rare events (e.g., hearing screening referral) have also been proposed, such as rare events logistic regression [22]. Utilizing rare events logistic regression (conducted using “relogit” in Stata 11), similar results were obtained to those shown in Supplemental Table 2, providing further support for the current conclusions.

Results of newborn hearing screening in HEU infants in SMARTT showed that 3.1% (45/1435) were referred for further testing, which is lower than the approximate 10% first referral rate previously reported in one statewide program in Rhode Island of all newborns [8]. This referral rate is also lower than that reported by Manfrediet al. [10] but similar to what Olusanya et al. [9] reported in children with HIV infection. Inconsistencies in referral rates are most likely a result of varying equipment used (i.e., OAE or AABR) and referral criteria across studies and even hospitals.

Because CMV-related hearing loss is not always present at birth, and can be progressive with time [11-14], the lack of an association with CMV in this subset at birth is not surprising. Longitudinal hearing testing is critical for these infants and needed to estimate the true risk of hearing loss with congenital CMV in PHEU children. The proportion of congenital CMV infection in HEU children in this study (1/22 = 4.5% in infants with hearing screening referral and 5/92 = 5.4% in infants who passed the hearing screening) was higher than that reported among children in the general US population (0.6%); it is consistent with rates reported in other HEU children [15-16,23]. There was no difference in the CMV infection rates by pass/refer results of the newborn hearing screening.

The Joint Committee on Infant Hearing [3] requires that newborns receive a hearing screening before leaving the hospital or at least by one month of age. The specific protocol for this screening is not standardized across hospitals but the universally accepted measures are OAEs and AABRs. The recording parameters for these measures as well as the pass/referral criteria can also vary. Because of this, one limitation of this study is that newborn screening protocols did vary across sites. Although OAE and AABR measures have different sensitivity and specificity rates for identifying hearing loss, the primary objective of this study was to identify HEU children who were referred for further hearing screening. Initial hearing screening data were obtained retrospectively and across various SMARTT sites within the U.S. and Puerto Rico so it was difficult to control for the various hearing screening protocols used. Another limitation is the lack of complete follow-up hearing data when a newborn was referred, or whether or not the newborn even had follow-up services after the initial referral.

Although HIV mother-to-child-transmission rates have declined dramatically in the U.S., [24] the number of HEU children will continue to increase. The decrease in HIV mother-to-child transmission is directly related to the expanded use of HAART during pregnancy. With the increasing number of ARVs and ARV combinations available for HIV treatment, it is important for future studies to further evaluate these potential adverse effects.

## Supplementary Material

Supp Table 1

Supp Table 2

## Figures and Tables

**Table 1:** Child and maternal characteristics by newborn hearing screening result, among infants with complete maternal antiretroviral exposure information^a^

Characteristic	Total (N = 1406)	Newborn screening result		*p - value*
		Pass (N = 1361)	Refer (N = 45)	
Female	682 (49%)	663 (49%)	19 (42%)	0.45
White	406 (30%)	394 (30%)^b^	12 (28%)	0.87
Hispanic	430 (31%)	416 (31%)	14 (31%)	1.00
**Birth Characteristics**				
Birth weight < 2.5 kg	251 (18%)	244 (18%)	7 (16%)	0.84
Gestational age < 37 weeks	272 (19%)	263 (19%)	9 (20%)	0.85
Mode of delivery – Cesarean	826 (59%)	799 (59%)	27 (60%)	1.00
**Maternal Characteristics at delivery**				
Mother < 25 years old	451 (32%)	437 (32%)	14 (31%)	1.00
Maternal viral load > 1000 copies/ml	174 (13%)	169 (13%)	5 (11%)	1.00
Maternal CD4 count < 200 cells/mm^3^	139 (10%)	133 (10%)	6 (14%)	0.44
**Maternal substance use during pregnancy**				
Tobacco use	250 (18%)	246 (18%)	4 (9%)	0.12
Alcohol use	123 (9%)	117 (9%)	6 (13%)	0.28
Illicit drug use	123 (9%)	118 (9%)	5 (11%)	0.59
Exposed to ototoxic medication *in utero*^c^	228 (16%)	216 (16%)	12 (27%)	0.06

a Twenty-nine infants did not have complete maternal ARV exposure data and are not included. All 29 of these passed the newborn screening.

b Percentages based on non-missing values. Seventy did not report race; 2 ethnicity; 11 birth weight and gestational age;; 12 mode of delivery; 11 mother's age at delivery; 28 mother's viral load at delivery; 30 mother's CD4 at delivery; 25 exposure to tobacco, alcohol or illicit drugs.

c Ototoxic medications include gentamicin, neomycin, streptomycin, amphotericin, erythromycin, vancomycin, ibuprofen, indomethacin, naproxen, hydrocodone, furosemide, tobramycin, and misoprostol.

## Abbreviations

CMV: Congenital Cytomegalovirus
SMARTT: Surveillance Monitoring of ART Toxicities
PHACS: Pediatric HIV/AIDS Cohort Study
ARV: Antiretroviral
HEU: HIV-Exposed Uninfected
aOR: Adjusted Odds Ratio
CI: Confidence Interval
HIV: Human Immunodeficiency Virus
HUU: HIV-Unexposed and Uninfected
HAART: Highly Active Antiretroviral Therapy
AABR: Automated Auditory Brainstem Response
OAE: Otoacoustic Emission
NRTI: Nucleoside Reverse Transcriptase Inhibitors
OR: Odds Ratio
