# Supplementary material for: Newborn Hearing Screenings in Human Immunodeficiency Virus-Exposed Uninfected Infants
Source: J AIDS Immune Res. Author manuscript; Available in PMC 2017 Apr 27. (PMC5407375)
Supplement: Supp Table 1 [file NIHMS842927-supplement-Supp_Table_1.docx]

**Supplemental Table 1. Exact logistic regression associations between child demographics and non-ARV maternal exposures and newborn hearing screening referral (or failed screening).**

| **Exposure** | **Odds Ratio** | **95% CI** | **P-value** |
| --- | --- | --- | --- |
| Female | 0.77 | (0.40, 1.46) | 0.48 |
| White | 0.88 | (0.41, 1.79) | 0.86 |
| Hispanic | 1.02 | (0.50, 2.01) | 1.00 |
| Birth weight < 2.5kg | 0.84 | (0.31, 1.92) | 0.84 |
| Gestational age < 37 weeks | 1.03 | (0.43, 2.22) | 1.00 |
| Small for gestational age | 1.16 | (0.35, 3.00) | 0.91 |
| APGAR score (1-minute) < 7 | 1.95 | (0.72, 4.55) | 0.19 |
| APGAR score (5-minute) < 7 | 1.24 | (0.03, 7.97) | 1.00 |
| Vaginal birth | 0.97 | (0.50, 1.84) | 1.00 |
| Mother <25 years at delivery | 0.94 | (0.46, 1.85) | 1.00 |
| Mother's VL >1000 cp/ml prior to deliver | 0.88 | (0.27, 2.29) | 1.00 |
| Mother's CD4 count <200 prior to deliver | 1.42 | (0.48, 3.48) | 0.56 |
| Tobacco use during pregnancy | 0.43 | (0.11, 1.21) | 0.14 |
| Alcohol use during pregnancy | 1.60 | (0.54, 3.92) | 0.41 |
| Illicit drug use during pregnancy | 1.29 | (0.39, 3.36) | 0.74 |
| Ototoxic med use during pregnancy | 1.93 | (0.89, 3.90) | 0.10 |
| Exposed to NSAID>6 days during pregnancy | 3.66 | (0.68, 12.69) | 0.13 |
| Exposed to aminoglycoside during pregnancy | 3.16 | (0.59, 10.82) | 0.17 |
| Exposed to aminoglycoside, furosemide | 2.99 | (0.74, 8.81) | 0.12 |
| Neonatal regimen of gentamicin | 1.51 | (0.63, 3.26) | 0.37 |
