# Supplementary material for: Newborn Hearing Screenings in Human Immunodeficiency Virus-Exposed Uninfected Infants
Source: J AIDS Immune Res. Author manuscript; Available in PMC 2017 Apr 27. (PMC5407375)
Supplement: Supp Table 2 [file NIHMS842927-supplement-Supp_Table_2.docx]

**Supplemental Table 2. Estimated associations between maternal ARV exposures and newborn hearing screening referral (or failed screening) using exact adjusted logistic regression.**

| **ARV exposure** | **Overall #**  **(% exposed) (N=1406)** | **Unadjusted odds ratio**  **(95% CI)** | **P-value** | **Adjusted odds ratio**^a^  **(95% CI)** | **P-value** |
| --- | --- | --- | --- | --- | --- |
| **Overall exposure** | | | | | |
| HAART/Triple NRTI | 1382 (98%) |  |  |  |  |
| HAART | 1250 (89%) | 2.74 (0.70, 23.59) | 0.21 | 2.67(0.68, 23.03) | 0.23 |
| NRTI | 1387 (99%) |  |  |  |  |
| Zidovudine | 925 (66%) | 0.85 (0.45, 1.68) | 0.71 | 0.87 (0.45, 1.71) | 0.76 |
| Lamivudine | 941 (67%) | 0.99 (0.51, 2.00) | 1.00 | 1.01 (0.52, 2.04) | 1.00 |
| Abacavir | 296 (21%) | 1.22 (0.55, 2.51) | 0.68 | 1.21 (0.54, 2.49) | 0.70 |
| Tenofovir | 602 (43%) | 0.81 (0.41, 1.54) | 0.59 | 0.79 (0.40, 1.52) | 0.56 |
| Emtricitabine | 565 (40%) | 0.82 (0.41, 1.57) | 0.63 | 0.81 (0.40, 1.55) | 0.60 |
| NNRTI | 154 (11%) | 1.52 (0.56, 3.53) | 0.43 | 1.46 (0.54, 3.39) | 0.49 |
| Efavirenz | 77 (5%) | 1.24 (0.24, 4.03) | 0.90 | 1.26 (0.24, 4.12) | 0.89 |
| Nevirapine | 60 (4%) | 0.50 (0.01, 3.06) | 0.84 | 0.48 (0.01, 2.97) | 0.80 |
| PI | 1160 (83%) | 1.16 (0.50, 3.11) | 0.91 | 1.17 (0.51, 3.16) | 0.89 |
| Atazanavir (ATV) | 343 (24%) | 1.74 (0.87, 3.37) | 0.12 | 1.84 (0.92, 3.56) | 0.09 |
| ATV-relogit^b^ |  | 1.74 (0.98, 3.27) | 0.07 | 1.81 (0.99,3.31) | 0.054 |
|  |  |  |  |  |  |
| Nelfinavir | 131 (9%) | 0.69 (0.13, 2.20) | 0.76 | 0.74 (0.14, 2.39) | 0.88 |
| Darunavir | 124 (9%) | 1.01 (0.26, 2.86) | 1.00 | 0.87 (0.22, 2.50) | 1.00 |
| Lopinavir/Ritonavir | 626 (45%) | 0.75 (0.38, 1.43) | 0.44 | 0.78 (0.40, 1.50) | 0.53 |
| II |  |  |  |  |  |
| Raltegravir | 86 (6%) | 1.97 (0.59, 5.20) | 0.27 | 1.92 (0.57, 5.08) | 0.29 |
|  |  |  |  |  |  |
| **1^st^ trimester exposure** | | | | | |
| HAART/Triple NRTI | 707 (50%) | 1.13 (0.60, 2.16) | 0.79 | 1.08 (0.57, 2.06) | 0.93 |
| HAART | 669 (48%) | 1.27 (0.67, 2.42) | 0.53 | 1.21 (0.64, 2.31) | 0.64 |
| NRTI | 724 (51%) | 1.08 (0.57, 2.06) | 0.92 | 1.02 (0.54, 1.95) | 1.00 |
| Zidovudine | 344 (24%) | 1.41 (0.68, 2.77) | 0.38 | 1.44 (0.70, 2.83) | 0.35 |
| Lamivudine | 387 (28%) | 1.63 (0.82, 3.12) | 0.17 | 1.61 (0.82, 3.10) | 0.18 |
| Abacavir | 131 (9%) | 1.23 (0.37, 3.18) | 0.82 | 1.14 (0.34, 2.97) | 0.94 |
| Tenofovir (TDF) | 375 (27%) | 0.41 (0.14, 1.00) | 0.05 | 0.39 (0.13, 0.94) | 0.03 |
| TDF-relogit^b^ |  | 0.46 (0.19, 1.07) | 0.063 | 0.43 (0.17, 1.05) | 0.053 |
|  |  |  |  |  |  |
| Emtricitabine (FTC) | 355 (25%) | 0.45 (0.15, 1.07) | 0.08 | 0.42 (0.14, 1.02) | 0.06 |
| FTC-relogit^b^ |  | 0.48 (0.21, 1.07) | 0.091 | 0.46 (0.19, 1.06) | 0.08 |
|  |  |  |  |  |  |
| NNRTI | 104 (7%) | 1.98 (0.67, 4.87) | 0.22 | 1.97 (0.66, 4.87) | 0.22 |
| Efavirenz | 60 (4%) | 1.63 (0.31, 5.36) | 0.60 | 1.73 (0.33, 5.73) | 0.55 |
| Nevirapine | 38 (3%) | 0.81 (0.02, 5.07) | 1.00 | 0.75 (0.02, 4.72) | 1.00 |
| PI | 588 (42%) | 1.12 (0.58, 2.12) | 0.83 | 1.07 (0.56, 2.04) | 0.93 |
| Atazanavir | 198 (14%) | 1.55 (0.65, 3.35) | 0.34 | 1.52 (0.63, 3.30) | 0.37 |
| Nelfinavir | 52 (4%) | 0.58 (0.01, 3.58) | 0.99 | 0.61 (0.01, 3.77) | 1.00 |
| Darunavir | 56 (4%) | 0.54 (0.01, 3.30) | 0.91 | 0.42 (0.01, 2.66) | 0.67 |
| Lopinavir/Ritonavir | 253 (18%) | 1.14 (0.48, 2.46) | 0.84 | 1.19 (0.50, 2.57) | 0.77 |
| II |  |  |  |  |  |
| Raltegravir | 45 (3%) | 2.24 (0.43, 7.48) | 0.34 | 2.04 (0.39, 6.87) | 0.41 |

^a^*Adjusted for maternal use of ototoxic medications and in utero tobacco exposure.*

^b^Relogit=rare events logistic regression from King and Zeng^22^

*HAART=highly active antiretroviral therapy; NRTI=nucleoside reverse transcriptase inhibitor; NNRTI=non-NRTI; PI=protease inhibitor; II=integrase inhibitor; CI=confidence interval.*
